# Expression of four cancer-testis antigens in TNBC indicating potential universal immunotherapeutic targets

**DOI:** 10.1007/s00432-023-05274-0

**Published:** 2023-08-23

**Authors:** Jie Xiao, Fengli Huang, Lin Li, Lianru Zhang, Li Xie, Baorui Liu

**Affiliations:** 1 Department of Oncology, Nanjing Drum Tower Hospital, Affiliated Hospital of Medical School, Nanjing University, Nanjing, China; 2 Department of Oncology, Nanjing Drum Tower Hospital Clinical College of Nanjing University of Chinese Medicine, Nanjing, 210008 China; 3 Department of Pathology, Nanjing Drum Tower Hospital, Affiliated Hospital of Medical School, Nanjing University, Nanjing, 210008 China

**Keywords:** Cancer-testis antigens, Target, Immunohistochemistry, Triple-negative breast cancer

## Abstract

**Objective:**

Immunotherapy is an attractive treatment for breast cancer. Cancer-testis antigens (CTAs) are potential targets for immunotherapy for their restricted expression. Here, we investigate the expression of CTAs in breast cancer and their value for prognosis. So as to hunt for a potential panel of CTAs for universal immunotherapeutic targets.

**Material and methods:**

A total of 137 breast cancer tissue specimens including 51 triple-negative breast cancer (TNBC) were assessed for MAGE-A4, MAGEA1, NY-ESO-1, KK-LC-1 and PRAME expression by immunohistochemistry. The expression of PD-L1 and TILs was also calculated and correlated with the five CTAs. Clinical data were collected to evaluate the CTA’s value for prognosis. Data from the K-M plotter were used as a validation cohort.

**Results:**

The expression of MAGE-A4, NY-ESO-1 and KK-LC-1 in TNBC was significantly higher than in non-TNBC (*P* = 0.012, *P* = 0.005, *P* < 0.001 respectively). 76.47% of TNBC expressed at least one of the five CTAs. Patients with positive expression of either MAGE-A4 or PRAME had a significantly extended disease-free survival (DFS). Data from the Kaplan–Meier plotter confirm our findings.

**Conclusions:**

MAGE-A4, NY-ESO-1, PRAME and KK-LC-1 are overexpressed in breast cancer, especially in TNBC. Positive expression of MAGE-A4 or PARME may be associated with prolonged DFS. A panel of CTAs is attractive universal targets for immunotherapy.

## Introduction

Breast cancer (BC) is considered the most common cancer worldwide, with approximately 2.3 million newly diagnosed in 2020 (Lei et al. 27). Breast cancer can be categorized into three major subtypes based on molecular markers, including hormone receptor-positive/HER-2-negative, HER-2-positive and triple-negative breast cancer. Triple-negative breast cancer (TNBC) is more likely to relapse and is not sensitive to endocrine therapy or anti-HER2 therapy. The median overall survival for metastatic triple-negative breast cancer is approximately 12–18 months (Waks and Winer 38). Given the success of immunotherapy in other tumor types(D'Angelo et al. 12; Hont et al. 21), it is essential to identify highly specific targets in TNBC.

Cancer-testis antigens (CTAs) are a type of protein that is exclusively detected in the testis but is aberrantly re-expressed in malignancy, especially high-grade and advanced-stage tumors (Albertsmeier et al. 4). Due to their unique expression profiles, CTAs are appealing targets for immunotherapy. Melanoma-associated antigen-A1 (MAGE-A1), Melanoma-associated antigen-A4 (MAGE-A4), New York Esophageal Squamous Cell Carcinoma-1 (NY-ESO-1) and Preferentially expressed Antigen in Melanoma (PRAME) expression in breast cancers attracted the most attention, but the expression rates reported varied widely (Adams et al. 1; Ademuyiwa et al. 2; Balafoutas et al. 5; Bandić et al. 6; Curigliano et al. 10; Raghavendra et al. 31) Kita-Kyushu lung cancer antigen-1 (KK-LC-1) is another immunogenic CTA considered to be overexpressed in TNBC at mRNA level (Chen et al. 9). A panel of CTAs, including MAGE-A4 and NY-ESO-1, is currently being used as multi-T cell targets for BC (Hoyos et al. 22). However, only patients with antigen-positive observed antigen-specific T cell amplification and induced disease stabilization. A breast cancer peptide vaccine derived from nine CTAs successfully induced an immune response against the vaccine (Dillon et al. 13). Thus, establishing a panel of universal CTAs with high specificity covering as many breast cancer patients as possible will have significant value.

The aim of this study was to detect the protein expression of the five CTAs (MAGE-A1, MAGE-A4, NY-ESO-1, PRAME, KK-LC-1) in breast cancer and find a possible universal target for breast cancer. The secondary objective was to assess the prognostic value of the five CTAs.

## Materials and methods

### Study population

A total of 137 archived breast cancer tissue specimens were obtained and detected at Drum Tower Hospital Medical School of Nanjing University. All of the enrolled patients have undergone curative resection, and all of the specimens were formalin-fixed and paraffin-embedded in 2018–2021. The histological slides were reviewed, and the diagnosis of breast cancer was confirmed by at least two pathologists. Among the 137 patients, there were 51 triple-negative breast cancer (TNBC) specimens and 86 other types of breast cancer (non-TNBC) specimens. All cases were examined for MAGE-A4, MAGE-A1, NY-ESO-1, KK-LC-1 and PRAME expression by immunohistochemistry (IHC).

Disease-free survival (DFS) was calculated from the time of the primary surgery to the occurrence of the first locoregional recurrence or distant metastasis.

### Tissue microarrays

Tissue microarrays (TMA) containing multiple human breast cancer tissues were obtained from the paraffin tissue blocks. Each sample point is 1.5 mm in diameter, and the thickness of the tissue section was 4 μm.

### Immunohistochemistry

Five cancer-testis antigens were analyzed by IHC, including MAGE-A4, MAGE-A1, NY-ESO-1, KK-LC-1 and PRAME. Hematoxylin and eosin (H&E) staining was performed to calculate TILs as well as to outline the tumor tissue location for TMA sampling. Slices were placed in Tris–EDTA buffer (pH = 9.0) for antigen repair in a microwave. Primary antibodies were used to probe the samples at 4 °C overnight. After being washed with PBS, the slices were incubated with a secondary antibody at room temperature for 30 min. Then, the slices were stained with diaminobenzidine and counterstained with hematoxylin. The following antibodies were used: NY-ESO-1 monoclonal antibody (1:1600, E978, Santa Cruz); MAGE-A1 monoclonal antibody (1:100, MA511338, Thermo Fisher); MAGE-A4 monoclonal antibody (1:200, Ab139297, Abcam); PRAME monoclonal antibody (1:16,000, Ab219650, Abcam); and KK-LC-1 monoclonal antibody (1:400, MA524711, Thermo Fisher). Archived normal human testis tissue was used as a positive control.

### Immunohistochemical scoring of CTAs

The protein expression of CTAs was estimated using a semiquantitative scoring system described as immunohistochemical score (Domfeh et al. 14). Both the degree of staining and the proportion of stained cells were assessed. The degree of staining (0 = negative staining,1 +  = weak staining, 2 +  = moderate staining, 3 +  = strong staining) is then multiplied by the proportion of stained cells (1 =  ≤ 10% positive, 2 =  > 10% and ≤ 50% positive, 3 =  > 50% and ≤ 80% positive and 4 =  > 80% positive). The final score was ranked as follows: 0 + IHC stain(≤ 3), 1 + IHC stain(> 3 and ≤ 6), 2 + IHC stain(> 6 and ≤ 9), 3 + IHC stain(> 9 and ≤ 12). 3 + IHC stain represented high expression, 2 + IHC stain represented moderate expression, 1 + IHC stain represented weak expression, and 0 + IHC stain represented negative expression.

### Evaluation of the expression of PD-L1 and TILs

Programmed death ligand 1 (PD-L1) on tumor cells (TC) and immune cells (IC) of the 137 breast cancer specimens was evaluated on SP142 assay by the department of Pathology. The assessment of tumor-infiltrating lymphocytes (TILs) was performed and calculated on H&E according to the previously described method (0 ~ 10% stromal TILs as low TILs, 10 ~ 50% as moderate TILs, 50 ~ 100% as high TILs) (Salgado et al. 34).

### Kaplan–Meier survival curve analysis

GSE16446 containing the information of 120 BRCA patients was assessed using the Kaplan–Meier plotter (kmplot.com/analysis) up to May 23, 2023. Log-rank P values were determined on the webpage.

### Statistical analysis

All the statistical analyses were performed using the SPSS 26 software package. CTA expression among groups was evaluated using the corrected chi-square test or Pearson chi square test. The Tau-b test was used for evaluation of the relationships between CTA expression and clinicopathological characteristics, PD-L1 and TILs. Survival probabilities were calculated by the Kaplan–Meir method. *P* values that were less than 0.05 were considered to be statistically significant. All tests were two-sided.

## Results

### Patient characteristics

From 2018 to 2021, a total of 137 pT1-3 pN0-3 M0 early breast cancer patients were enrolled. The descriptive characteristics are tabulated (Table 1). Tumor stages were pT1-2 for 132 patients and pT3-4 for 5 patients. Nodal invasion was detected in 77 patients, while 60 were examined as pN0. According to the WHO grades, 73 tumors were classified as grade III, 54 were grade II, and 10 were grade I (Table 1).

**Table 1:** Baseline pathological characteristics of all patients

	TNBC		Non-TNBC	
Gender				
Female	51	100.0%	86	100.0%
Male	0	0.0%	0	0.0%
Age				
> 51	31	60.8%	49	57.0%
≤ 50	20	39.2%	37	43.0%
WHO grade				
I	0	0.0%	10	11.6%
II	11	21.6%	43	50.0%
III	40	78.4%	33	38.4%
*AJCC 8*^*th*^				
T				
I–II	50	98.0%	82	95.3%
III–IV	1	2.0%	4	4.7%
N				
–	27	52.9%	33	38.4%
+	24	47.1%	53	61.6%
M				
0	51	100.0%	85	98.8%
1	0	0.0%	0	0.0%
Unknown	0	0.0%	1	1.2%
Neuroinvasion				
–	48	94.1%	72	83.7%
+	3	5.9%	14	16.3%
Lymphovascular invasion				
–	30	58.8%	56	65.1%
+	21	41.2%	30	34.9%
ER				
–	51	100.0%	12	14.0%
+	0	0.0%	74	86.0%
PR				
–	51	100.0%	23	26.7%
+	0	0.0%	53	61.6%
Her-2				
–	51	100.0%	60	69.8%
+	0	0.0%	26	30.2%

### Immunohistochemical expression of CTAs in breast cancer

**Fig. 1:**
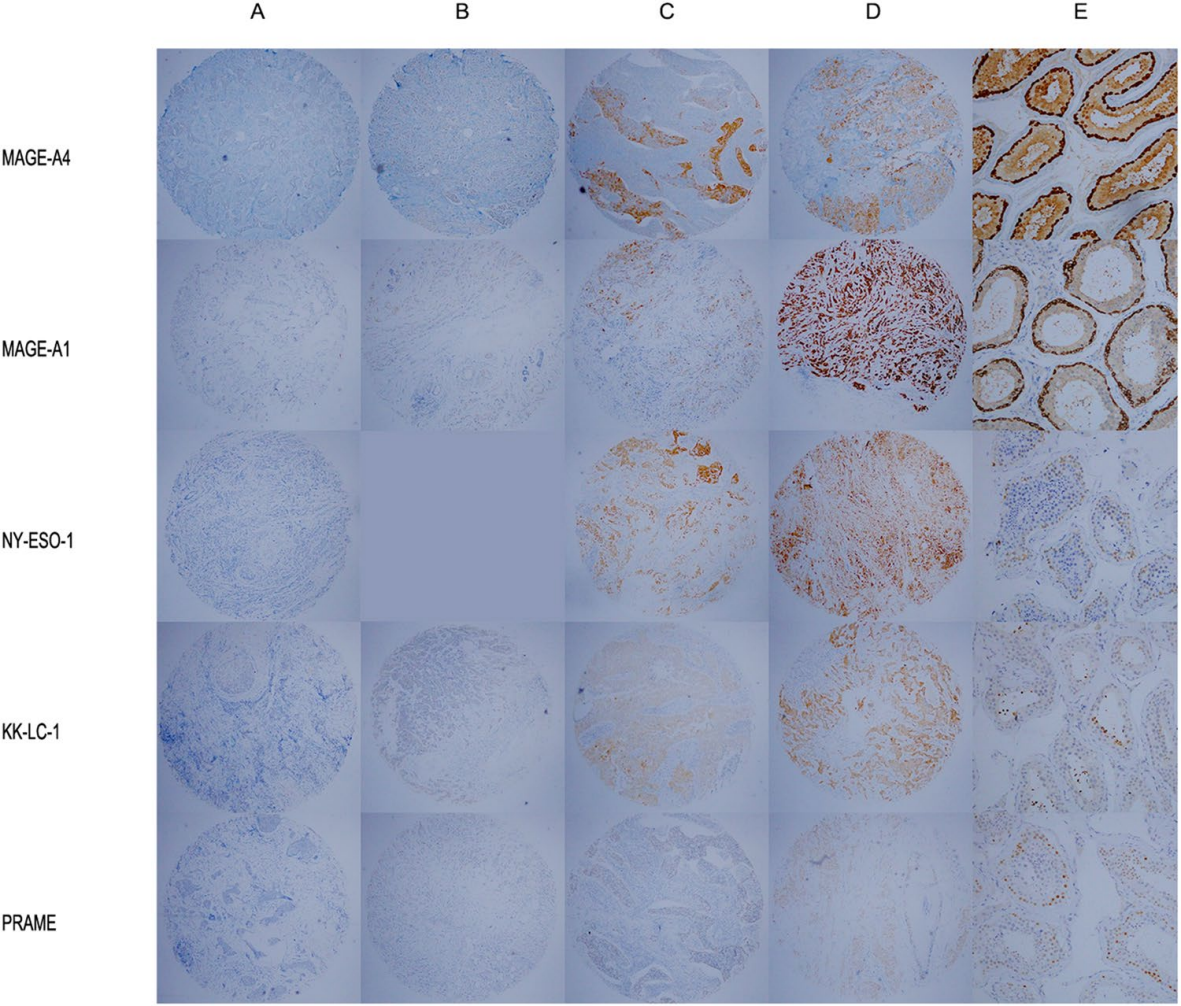
Examples of CTAs IHC staining. **A** 0 + IHC staining (× 4); **B** 1 + IHC staining (× 4); **C** 2 + IHC staining (× 4); **D** 3 + IHC staining (× 4); **E** positive control of testicular tissue (× 20)

**Fig. 2:**
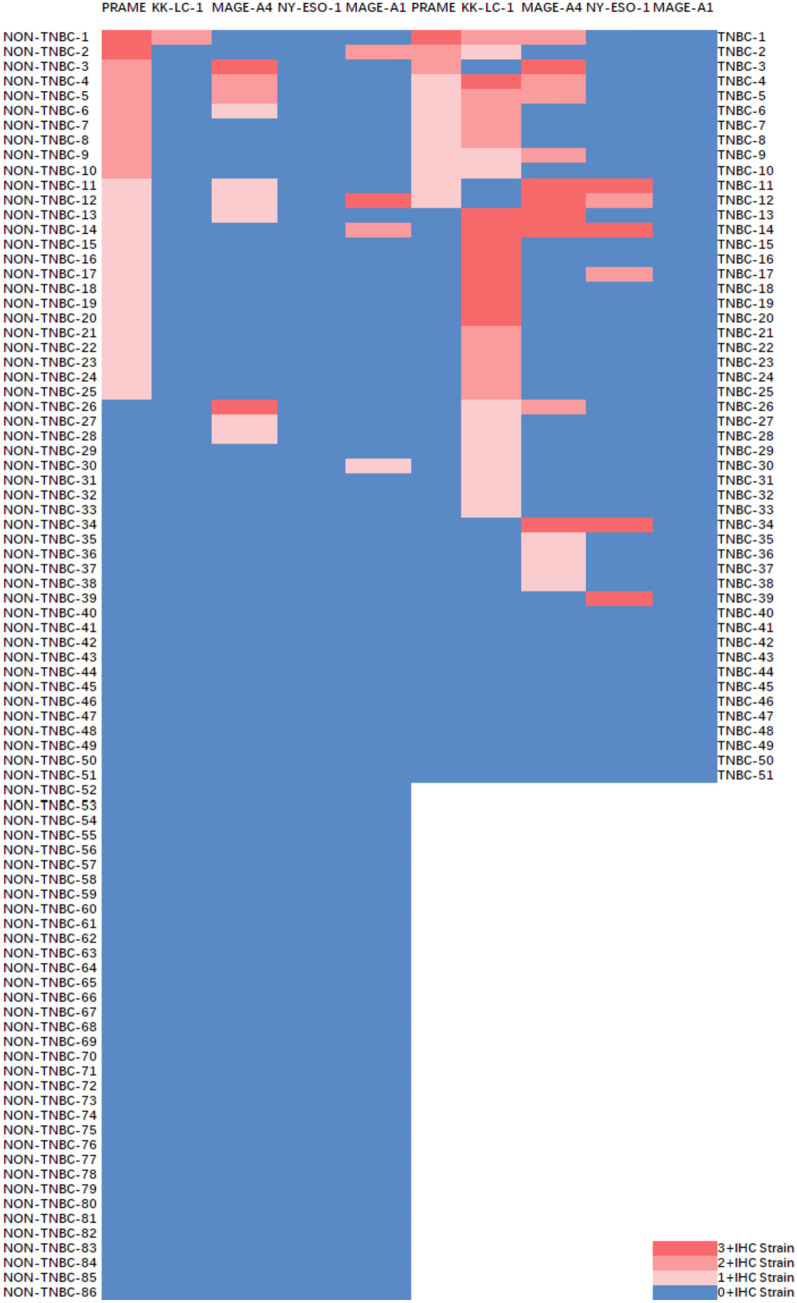
Heat maps of the CTAs expression in TNBC and non-TNBC. Individual antigen expression of PRAME, KK-LC-1, MAGE-A4, NY-ESO-1 and MAGE-A1

Expression levels of MAGE-A1, MAGE-A4, NY-ESO-1, KK-LC-1 and PRAME were examined by IHC. The expression pattern was heterogeneous, ranging from 0 + to 3 + (Fig. 1). The expression of CTAs in TNBC and non-TNBC is summarized and analyzed in Table 2, and the heat map shows the immunohistochemical scoring for each one of the samples (Fig. 2). MAGE-A4 expression (immunohistochemical scoring > 0) was found in 15 (29.41%) TNBCs, but only in 10 (11.63%) non-TNBCs (*P* = 0.012). NY-ESO-1 was documented in 6 (11.76%) in TNBC while 0 in non-TNBC (*P* = 0.005). KK-LC-1 was expressed in 30 (58.82%) in TNBC but only in 1 (1.16%) in non-TNBC (*P* < 0.001). MAGE-A1 and PRAME were non-significantly different between the two groups with 2.92% and 27.01% in breast cancer, respectively. All TNBC tissues did not express MAGE-A1 and only four non-TNBC expressed MAGE-A1. Among the five CTAs, 49.64% of breast tumors expressed at least one. There were 76.47% TNBC tumors expressing at least one CTA compared to 33.72% in non-TNBC (*P* < 0.001). There were 7 TNBC tumors that expressed more than 3 CTAs, compared to only one case of non-TNBC expressing three CTAs at the same time (*P* = 0.008). Overall, the expression frequency of CTAs in TNBC was higher than that detected in non-TNBC. A panel of four CTAs (MAGE-A4, NY-ESO-1, KK-LC-1 and PRAME) covers 76.47% of TNBC tumors at the protein level.

**Table 2:** Expression of the five CTAs in TNBC and non-TNBC tumors

	BC		TNBC		Non-TNBC		*P*
	*N*	%	*N*	%	*N*	%	
MAGE-A4	25	18.25%	15	29.41%	10	11.63%	0.012*
MAGE-A1	4	2.92%	0	0.00%	4	4.65%	0.299
NY-ESO-1	6	4.38%	6	11.76%	0	0.00%	0.005**
KK-LC-1	31	22.63%	30	58.82%	1	1.16%	< 0.001***
PRAME	37	27.01%	12	23.53%	25	29.07%	0.553
CTA ≥ 1	68	49.64%	39	76.47%	29	33.72%	< 0.001***
CTA ≥ 2	27	19.71%	17	33.33%	10	11.63%	0.003**
CTA ≥ 3	8	5.84%	7	13.73%	1	1.16%	0.008**

*P* values were calculated by two-tailed unpaired Student’s *t* tests. *P < 0.05, **P < 0.01, ***P < 0.001

### Correlation between CTAs expression and biomarkers for immunotherapy

The relationship between every two CTA expressions was compared. MAGE-A4 and NY-ESO-1 protein expression levels demonstrated a positive correlation (*r* = 0.303 *p* < 0.001). There was also a correlation between the expression of MAGEA4 and PRAME (*r* = 0.303 *p* < 0.001) (Table 3). We further provide insight into the co-expression between CTA expression, PD-L1 expression and TILs. Unfortunately, we failed to find any statistically significant relationship between CTAs and PD-L1/TILs.

**Table 3:** Correlation among CTAs and PD-L1/TILs

	MAGE-A4	MAGE-A1	NY-ESO-1	KK-LC-1	PRAME	PD-L1 TC	PD-L1 IC	TILs
MAGE-A4	*R* = 1.000	*R* = 0.020, *P* = 0.812	*R* = 0.303, *P* < 0.001***	*R* = 0.081, *P* = 0.314	*R* = 0.303, *P* < 0.001***	*R* = 0.226, *P* = 0.007**	*R* = 0.094, *P* = 0.257	*R* = − 0.023, *P* = 0.784
MAGE-A1		*R* = 1.000	*R* = − 0.036, *P* = 0.665	*R* =− 0.089, *P* = 0.279	*R* = 0.185, *P* = 0.025*	*R* = 0.036, *P* = 0.669	*R* = − 0.058, *P* = 0.497	*R* = 0.066, *P* = 0.438
NY-ESO-1			*R* = 1.000	*R* = 0.077, *P* = 0.35	*R* = 0.013, *P* = 0.871	*R* = − 0.003, *P* = 0.968	*R* = 0.075, *P* = 0.379	*R* = 0.162, *P* = 0.058
KK-LC-1				*R* = 1.000	*R* = 0.051, *P* = 0.525	*R* = 0.229, *P* = 0.006**	*R* = 0.194, *P* = 0.019*	*R* = 0.094, *P* = 0.258
PRAME					*R* = 1.000	*R* = 0.233, *P* = 0.005**	*R* = − 0.015, *P* = 0.854	*R* = 0.005, *P* = 0.950

*P < 0.05, **P < 0.01, ***P < 0.001

### Expression of CTAs indicates higher grade of pathology

Our studies have shown that MAGE-A4, MAGE-A1, NY-ESO-1 and KK-LC-1 expression was associated with WHO grades (*P* = 0.041, *P* = 0.023, *P* = 0.023 and *P* < 0.001, respectively). Positive expression of MAGE-A4, NY-ESO-1 and KK-LC-1 suggested a higher histological grade of breast cancer (Table 4). However, there was no significant relationship between the CTA expression and other clinical factors, including age, lymph node status, tumor size, lymphovascular invasion, or neuroinvasion.

**Table 4:** CTAs expression in subgroups

Characteristic	Patients	MAGE-A4		*P*	MAGE-A1		*P*	NY-ESO-1		*P*	KK-LC-1		*P*	PRAME		*P*
		+	–		+	–		+	–		+	–		+	–	
Age				0.185			0.748			0.198			0.328			0.670
> 51	40	18	62		2	78		5	75		16	64		21	59	
≤ 50	57	7	50		2	55		1	56		15	42		16	41	
WHO grade				0.041*			0.023*			0.023*			< 0.001***			0.935
I	10	0	10		1	9		0	10		0	10		2	8	
II	54	8	46		3	51		0	54		4	50		16	38	
III	73	17	56		0	73		6	67		27	46		19	54	
T				0.980			0.694			0.627			0.224			0.653
I–II	132	24	108		4	128		6	126		31	101		36	96	
III–IV	5	1	4		0	5		0	5		0	5		1	4	
N				0.501			0.430			0.260			0.081			0.770
–	60	12	48		1	59		4	56		18	42		17	43	
+	77	13	64		3	74		2	75		13	64		20	57	
Neuroinvasion				0.814			0.443			0.343			0.215			0.684
–	118	22	96		4	114		6	112		29	89		32	86	
+	19	3	16		0	19		0	19		2	17		5	14	
Lymphovascular invasion				0.468			0.586			0.820			0.215			0.487
–	85	14	71		2	83		4	81		17	68		24	61	
+	52	11	41		2	50		2	50		14	38		13	39	

*P* values were calculated by two-tailed unpaired Student’s *t* tests. *P < 0.05, ***P < 0.001

### Prognostic effect of CTA expression

**Fig. 3:**
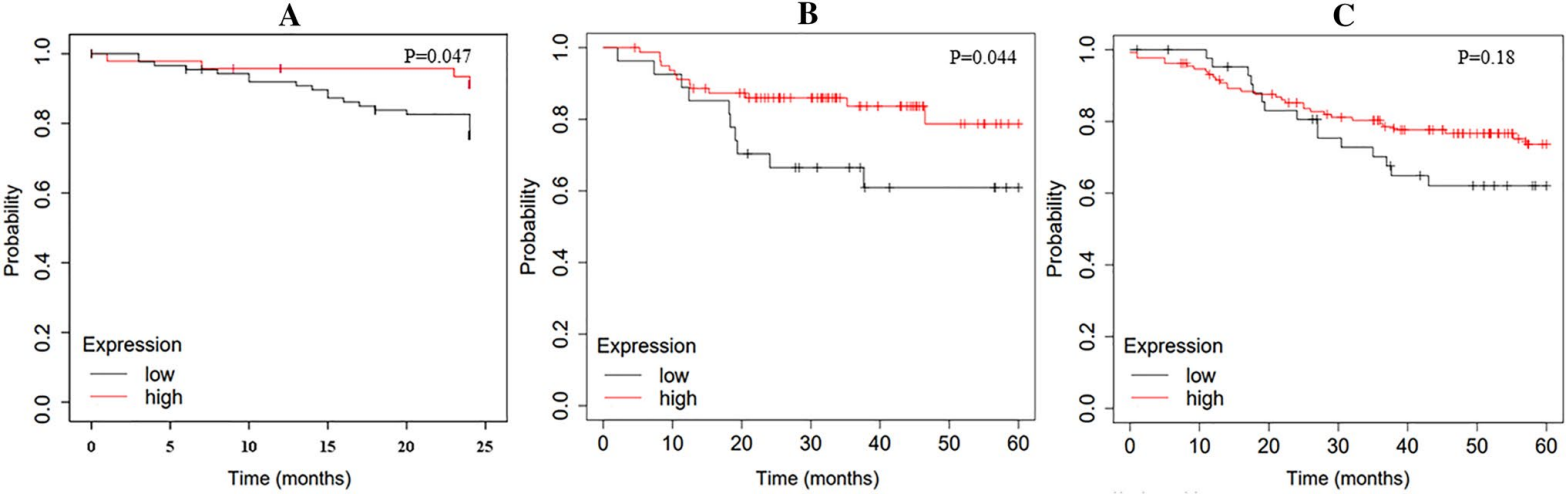
Disease-free survival (DFS) of patients with breast cancer based on CTA expression status. **A** Patients positive for MAGE-A4 or PRAME (high) show significantly better (*P* = 0.047) DFS than patients with negative expression status(low). **B** Patients with breast cancer from GSE16446 based on MAGE-A4 and PRAME RNA expression status show the high group owned an improved DFS (*P* = 0.044). **C** TNBC patients with a high RNA expression of any of the 4 CTAs from Kaplan–Meier plotter tended to have a longer DFS (57 months vs 30.42 months).

All 137 patients were followed up for a median of 27.9 months, and no single CTA has been found to be related to disease-free survival. Further analysis identified that positive expression of MAGE-A4 or PRAME significantly extended DFS (23.13 months vs 21.83 months *P* = 0.047, Fig. 3A). No significant correlation with other prognostically relevant markers was observed in this subset. Data generated by Kaplan–Meier plotter (kmplot.com) also confirm our findings (Fig. 3B). The expression on the mRNA level of either MAGE-A4 or PRAME may indicate a longer DFS (*P* = 0.044). Moreover, 176 TNBC patients with high RNA expression for either of the 4 CTAs (all except for MAGE-A1) from the Kaplan–Meier plotter tended to have a longer DFS (57 months vs 30.42 months) (Fig. 3C). Therefore, these results motivated us to further evaluate CTAs as a potential prognostic biomarker in TNBC.

## Discussion

In this study, CTAs expression was assessed in 137 breast cancer samples by IHC. Our results showed that MAGE-A4, NY-ESO-1 and KK-LC-1 were overexpressed in TNBC at the protein level, which is consistent with the results of several other studies (Chen et al. 9; Curigliano et al. 10; Kondo et al. 24; Raghavendra et al. 31). In contrast, we did not find a significantly higher expression of PRAME in TNBC as reported in the previous study (Curigliano et al. 10). Such differences can be partly explained by the heterogeneity of tumors and the limited size of the TMA sample. The low detection of MAGE-A1 differed from the 31% positive reported by Fujie et al. (18). This may be caused by differences in gene expression at the RNA and protein levels. The expression of NY-ESO-1 was found to be correlated with a higher level of tumor-infiltrating lymphocytes (Lee et al. 26), which was not confirmed in our study, probably due to the few positive cases. Further research is required because the expression of the CTAs was only identified at the protein level. An additional detection route for CTAs expression would be interesting.

Critical factors that affect the treatment efficacy of cancer immunotherapy comprise issues like the nature of tumor antigens, the quality of immune responses, and the immune microenvironment (Benvenuto et al. 7). In terms of enhancing the incidence and extent of the response while decreasing the likelihood of cancer progression in TNBC patients, ICBs have demonstrated potential efficacy when combined with chemotherapy (Qi et al. 30). Major challenges associated with ICBs are the primary resistance with only a few patients responding to this treatment, and the secondary resistance which resulted in a few patients experiencing long-lasting benefits as a consequence of the treatment (Dammeijer et al. 11). Therapeutic cancer vaccines demonstrated a potential effect to reverse resistance against ICBs in tumors like melanoma and lung cancer, which indicates a promising treatment model that combines both ICBs and vaccines (Fourcade et al. 17; Hannani et al. 20). Adoptive cell therapy is another important type of targeted immunotherapy. T-cell receptor (TCR)-transduced T cells targeting KRAS G12D also demonstrated great potential in tumor control (Leidner et al. 28). An antigen targeted T cell therapy against CTAs demonstrates promising results in antigen positive breast cancer patients (Hoyos et al. 22). Thus, a panel of universal tumor specific targets with high specificity and immunogenicity covering as many breast cancer patients as possible will have great application prospects.

CTAs expression is normally mainly restricted to the testis and placenta (Fan et al. 16; Simpson et al. 36). Among these CTAs, MAGE-A4 and NY-ESO-1 were most successfully applied for their excellent immunogenicity (Ebert et al. 15; Maxfield et al. 29; Saito et al. 33). The immunogenicity of PRAME has long been found in melanoma (LaVoy et al. 25), while the immunogenic KK-LC-1 peptide restricted by HLA-B62 and HLA-A2 was discovered in lung adenocarcinoma (Fukuyama et al. 19). The tumor specificity and immunogenicity of CTAs warrant us to explore the feasibility of designing a universal panel of CTAs to be immunotherapy targets. Our results indicated that 76.47% of TNBC expressed at least one CTA from MAGE-A4, NY-ESO-1, KK-LC-1 and PRAME.

Besides, CTAs are also found to be potential biomarkers for prognosis. A previous study suggests that PRAME positivity may be associated with a lower risk of early metastasis of TNBC (See et al. 35). Sun et al. found PRAME inhibits the growth of breast cancer in a mouse model (Sun et al. 37). NY-ESO-1 is associated with a better prognosis in 1234 TNBC samples (Lee et al. 26). Mirko Samija et al. observed patients with MAGE-A4-positive owned a significantly longer survival in women diagnosed as invasive ductal breast cancer (Bandić et al. 6). Few researches have been conducted on KK-LC-1, and a bioinformatic analysis showed that high KK-LC-1 expression was associated with poorer overall survival (Chen et al. 9). Our results did not suggest a statistically significant association of these CTAs with prognosis, probably due to the fact that the follow-up was not long enough. However, PRAME combined with MAGE-A4 seems to be inversely correlated with recurrence. Our study also showed a trend toward better DFS in patients with any CTAs positive for MAGE-A4, NY-ESO-1, KK-LC-1 and PRAME. No studies have proven CTAs expression as a driver event in tumorigenesis. The positive expression of CTAs may be the result of coordinated gene expression as it signals poor tumor differentiation (Brightwell et al. 8; Curigliano et al. 10). This is consistent with our finding that CTAs expression is associated with a higher pathological grade.

The suppressive cells and cytokines in the tumor microenvironment, as well as checkpoint molecules expressed on the tumor and infiltrating immune cells, eventually affect the long-term survival of the tumor (Hui and Chen 23). PD-L1 positivity is a recognized biomarker for current immunotherapy in clinical practice (Ahn and Kim 3; Reis et al. 32). Here, we attempted to explore the association between CTAs expression and PD-L1 in the tumor microenvironment and failed to documented a positive interlink. We further explored the association between CTAs expression and TILs, but unfortunately, no significant relationship was observed.

It could prospect that further research will focus on vaccines covering the epitopes of the four CTAs. On the other hand, universal TCR-T cell therapy targeting the four CTAs could also be a candidate treatment for TNBC.

## Conclusion

In this study, we detected the five CTAs protein expression in BC and found that TNBC had a higher frequency of expression. On top of this, we identified a panel of four CTAs, MAGE-A4, NY-ESO-1, PRAME and KK-LC-1, expressed in 76.47% of TNBC tumors. Breast cancer patients with positive expression for either MAGE-A4 or PRAME have extended disease-free survival. It could be presumed that vaccines or adoptive immune cells targeting the four CTAs may shed new light on the future TNBC treatment.

## Data Availability

The datasets generated and analyzed during the current study are available in K-M plotter (https://kmplot.com/).
